# Jones Fractures in Sumo Wrestlers: Three Case Reports

**DOI:** 10.1155/2019/9051327

**Published:** 2019-10-24

**Authors:** Takashi Hoshino, Tomohiko Tateishi, Tsuyoshi Nagase, Arata Yuki, Teruhiko Nakagawa, Masamitsu Tsuchiya

**Affiliations:** ^1^Department of Orthopaedic Surgery, Doai Memorial Hospital, 2-1-11 Yokoami, Sumida-ku, Tokyo 130-8587, Japan; ^2^Department of Orthopaedic Surgery, Tokyo Medical and Dental University Hospital of Medicine, 1-5-45 Yushima, Bunkyo-ku, Tokyo 113-8519, Japan; ^3^Department of Orthopaedic Surgery, Soka Municipal Hospital, 2-21-1, Soka, Soka City, Saitama 340-8560, Japan

## Abstract

Jones fractures sometimes occur in athletes and are known to have complications, such as nonunion, delayed union, and recurrence, even with treatment. We describe three cases of Jones fractures in sumo wrestlers with treatment-related difficulties. All patients discontinued treatment at their own discretion. The two conservative cases had nonunion or delayed union, and the operative case had a broken screw. However, all patients continued sumo wrestling, with little impact on their careers. The risk factors of Jones fractures in sumo wrestling may be heavy weight, and training or competition characteristics unique to sumo wrestling. In cases of a complete Jones fracture, operative treatment is most commonly selected, as the risk for nonunion or refractures is less than that for conservative treatment. However, in the case of sumo wrestlers, there are risks of infection and problems with treatment compliance. As taking a rest may result in a lowered rank, completing a sufficient duration of treatment is difficult. Treatment is difficult and controversial in sumo wrestlers; all three patients discontinued treatment of their own accord. These cases suggest that it is important to thoroughly inform sumo wrestlers of the treatment options, and to decide the most appropriate treatment method for each patient.

## 1. Introduction

Stress fractures in the fifth metatarsal at the transition of the epiphysis to the metaphysis, known as Jones fractures, were first reported by Sir Robert Jones in 1902 [1]. Jones fractures sometimes occur in athletes, especially in soccer, basketball, and football players [2–4]. Jones fractures are generally treated either conservatively or operatively [5, 6]. Conservative treatment often includes the use of a sole plate and fixation with a cast. Operative treatment often involves screw fixation and bone transplantation [7, 8]. However, even after treatment, nonunion, delayed union, or recurrence may occur [9, 10]. As a result, it may take time for the injured athlete to return to sports [11, 12].

Sumo wrestling is a traditional sport in Japan, with a 2000-year history. Professional sumo wrestling consists of six ranked divisions [13]. Except for the Mawashi (belt), sumo wrestlers are naked and they use their full strength during one-on-one contact. They are known to be at high risk of injury due to their large size and heavy weight [14–16]. Tsuchiya reported on 5094 injuries in 1425 sumo wrestlers that occurred over a period of 27 years. The most frequent location of injury was the lower extremities (51.2%), followed by the trunk (26.3%), upper extremities (21.3%), and others (1.1%), demonstrating that sumo wrestlers are susceptible to lower extremity injuries [17, 18]. However, as sumo wrestlers may lose a rank by taking time to rest and heal from injuries, and they may not be able to compete during treatment, treatment compliance is difficult.

Here, we report on three cases of Jones fractures in sumo wrestlers with treatment-related difficulties. As there are few reports on traumas related to sumo wrestling, these rare case reports markedly extend the literature. Written informed consent was obtained from all participants included in this study, and this study meets the ethical standards of the journal [19].

## 2. Case Reports

### 2.1. Case 1

**Figure 1:**
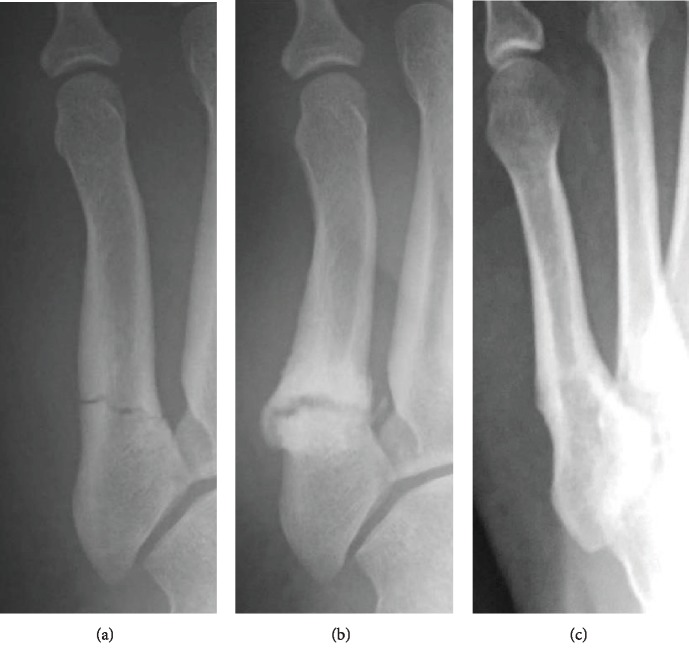
Case 1: anteroposterior radiographs of a 19-year-old sumo wrestler with a Jones fracture (a–c). At 9 months after conservative treatment, nonunion is observed on radiography (b). However, 4 years later, bone union is observed (c).

The patient was a 19-year-old, male, third-ranked sumo wrestler (height, 185 cm; weight, 150 kg). He twisted his left foot during sumo training and felt pain. Two weeks later, he first visited our hospital. He complained of pain in the outside of his left foot. Radiography showed fractures of the proximal epiphysis of the fifth metatarsal bone (Figure 1(a)). At the fracture site, thickening of the periosteum was observed. His diagnosis was Jones fracture; we performed conservative treatment with cast fixation. One month after his first visit, he discontinued visiting the hospital and returned to sumo wrestling at his own discretion. Nine months later, he injured his anterior cruciate ligament and returned to the hospital. Radiography showed nonunion of the Jones fracture (Figure 1(b)), but he did not go down in rank after the fracture. Four years after the injury, bone union was observed (Figure 1(c)).

### 2.2. Case 2

**Figure 2:**
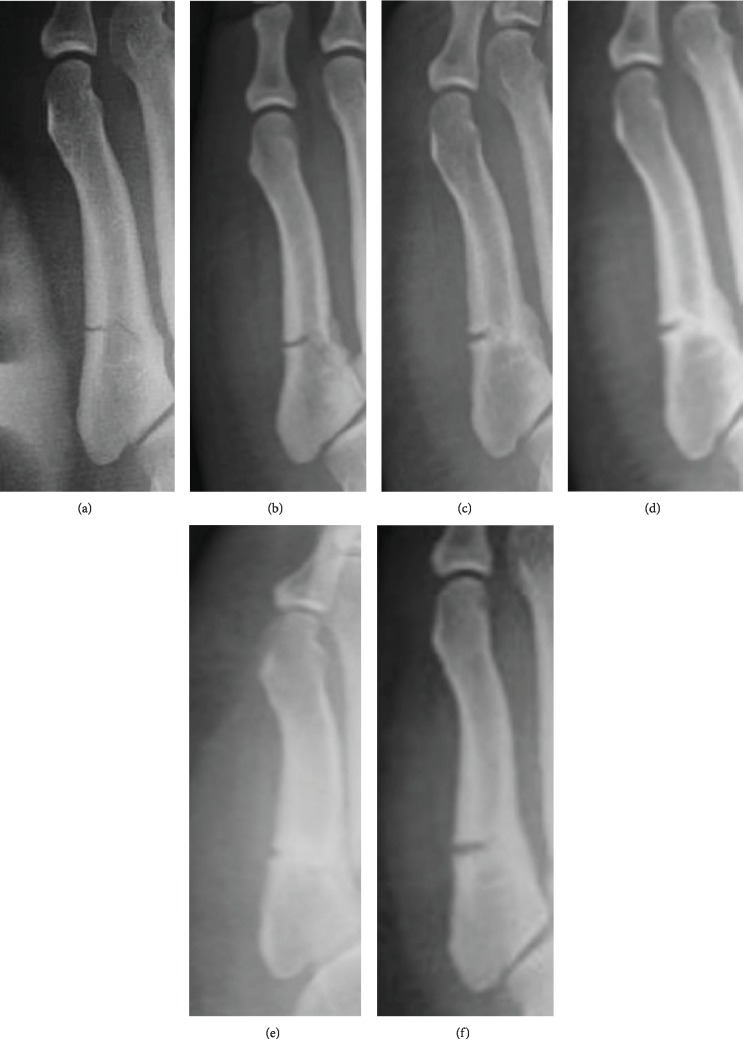
Case 2: anteroposterior radiographs of a 22-year-old sumo wrestler with a Jones fracture (a–f). Radiography shows gradual bone union at 2 weeks (b), 10 weeks (c), 3 months (d), and 4 months (e) after conservative treatment. However, at 6 months after treatment, the foot was refractured (f).

The patient was a 22-year-old, male, third-ranked sumo wrestler (height, 173 cm; weight, 136 kg). He was injured during sumo training. Two days after the injury, he visited our hospital. Radiography revealed fractures of the proximal epiphysis of the fifth metatarsal bone and thickening of the periosteum (Figure 2(a)). We diagnosed Jones fracture and performed conservative treatment with cast fixation for three weeks. Subsequently, we removed the cast and put on a brace. Radiography showed gradual bone union; thus, his amount of training gradually increased (Figures 2(b)–2(e)). Six months after the injury, his foot was refractured (Figure 2(f)). We started treatment with Low-Intensity Pulsed Ultrasound (LIPUS) in combination with the brace [20], but he discontinued treatment at his own discretion. However, he continued to sumo wrestle.

### 2.3. Case 3

**Figure 3:**
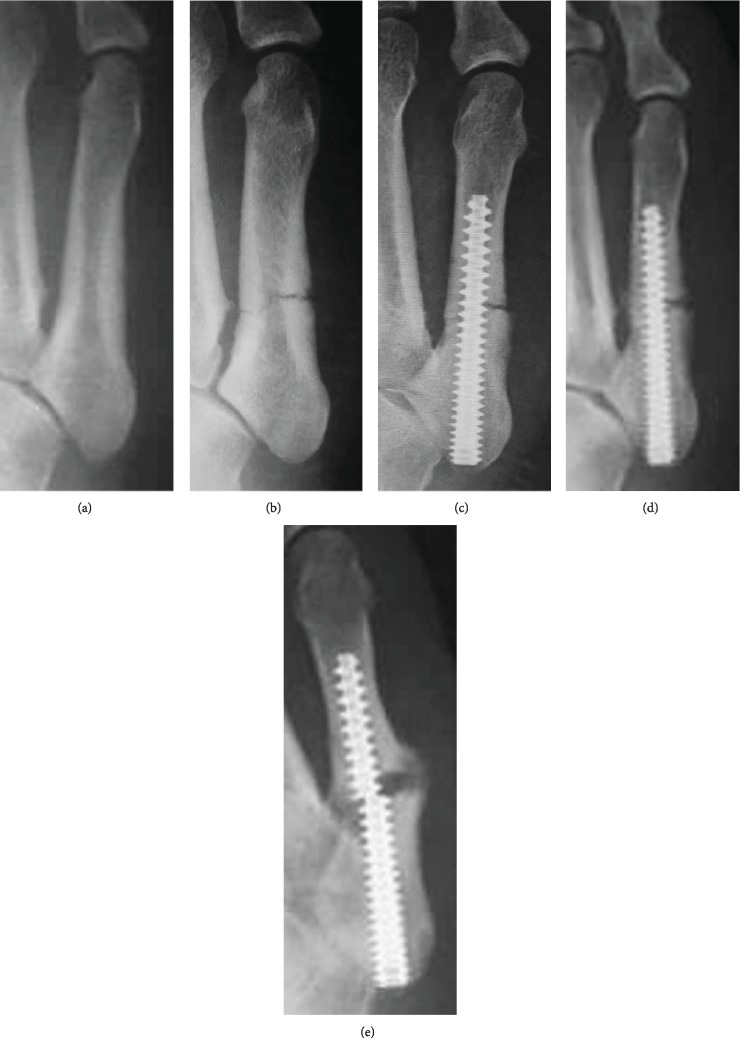
Case 3: anteroposterior radiographs of a 28-year-old sumo wrestler taken when he first visited another hospital (a) and 1 week later (b). We treated the Jones fracture operatively at 2 weeks after the first visit (c). Radiographs taken 4 weeks postoperatively are shown (d). At 4 months after operation, a screw broke (e).

The patient was a 28-year-old, male, third-ranked sumo wrestler (height, 175 cm; weight, 133 kg). He felt pain in his right foot during a tournament. After the tournament, the pain in his right foot became worse and he visited another clinic. Radiography did not show a fracture, but the periosteum was thickened; thus, there was a possibility of fatigue fractures (Figure 3(a)). One week later, a reexamination revealed a fracture of the proximal epiphysis of the fifth metatarsal bone (Figure 3(b)). He was referred to our hospital with a diagnosis of Jones fracture. After consulting with him and his director about treatment options, he strongly desired operative treatment. We performed the operation 2 weeks after his first visit to the other clinic (Figure 3(c)). He underwent Acutrak headless compression screw fixation without bone transplantation. After the operation, he underwent rehabilitation with full weight bearing within pain. We started treatment with LIPUS on the fourth postoperative day. He gradually increased his training level, but returned to a tournament without permission one month postoperatively (Figure 3(d)). Although he had pain in his right foot, he continued to sumo wrestle. However, a screw broke four months postoperatively (Figure 3(e)). Although he had nonunion, he continued to sumo wrestle.

## 3. Discussion

We experienced three cases of Jones fracture in sumo wrestlers, two of which were treated conservatively and one was treated operatively. All three patients discontinued treatment at their own discretion and had nonunion or delayed union.

Jones fractures are known to occur often in athletes who perform pivoting sports, such as soccer, football, and basketball. Repeated stress on the outside of the foot can cause stress fractures [21]. Various risk factors for Jones fractures have been reported, including physical features and external factors. For example, Raikin et al. reported that many patients with Jones fractures have evidence of varus hindfoot alignment [22]. Furthermore, Saita et al. reported that the restriction of the hip internal rotation is associated with an increased risk of developing a Jones fracture [23]. Other external factors, such as shoes and artificial turf, have been reported [3]. However, these reports comprise mainly soccer players. In the case of sumo wrestlers, the risk factors for Jones fractures may be heavy weight, and training or competition characteristics unique to sumo wrestling.

**Figure 4:**
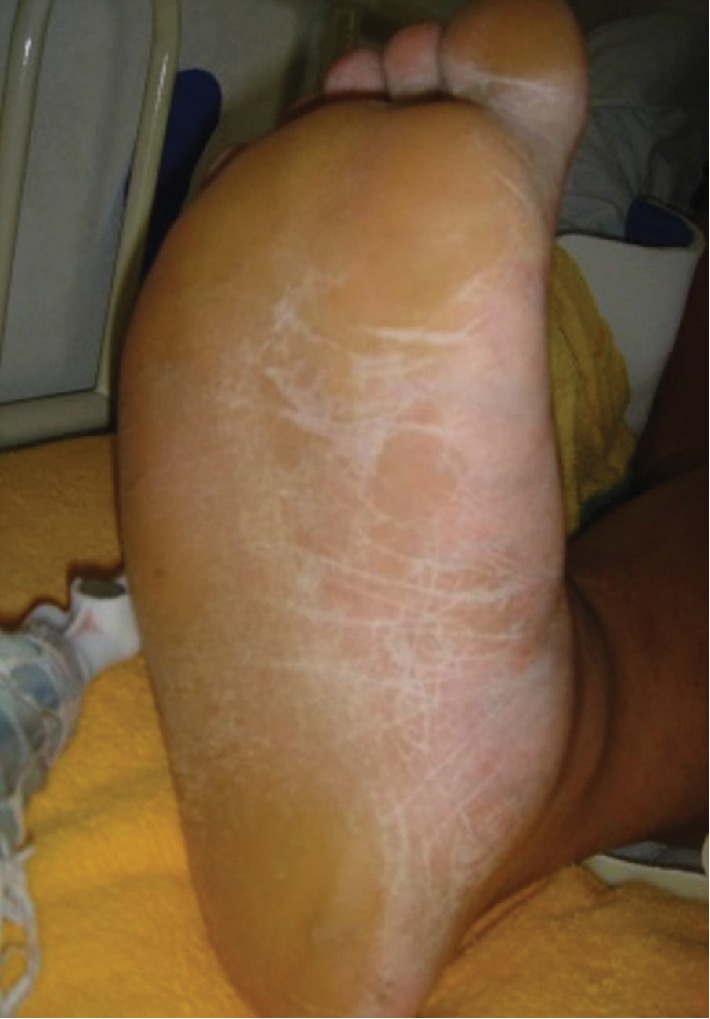
A Sumo wrestler's foot. The sole of the sumo wrestler is thickened around outside and is keratinized.

Concern exists regarding the burden on the sole of the foot during Suriashi and Shiko training. In Suriashi training, the sole of the foot is kept off the ground while the posture of the middle and low back is maintained. In Shiko, a sumo wrestler lifts both feet high alternately, and puts his hand on the knee, placing pressure toward the ground [13]. Furthermore, it is expected that a load on the sole of the foot is imposed when enduring collisions. The soles of sumo wrestlers (Figure 4) are typically thickened around the outside, suggesting that a load is consistently applied to the outside of the foot. In addition, since the dohyo is made of soil and is very hard, the influence of the ground should be considered.

Conservative or operative treatment can be selected for treating Jones fractures. Tateishi et al. reported that incomplete Jones fractures treated with LIPUS may heal without taking a break from practice [24]. However, in cases of a complete fracture, operative treatment is most commonly selected. Previous reviews have indicated that the risk of nonunion or refractures is less with operative treatment than with conservative treatment [11, 12, 25]. Additionally, good postoperative clinical outcomes have been reported [7, 8]. However, it is important to consider which is better for the sumo wrestler: conservative treatment or operative treatment? Sumo wrestling is a contact sport that is performed barefoot in the dohyo, without wearing anything except the Mawashi. As shown in Figure 4, the sole of the sumo wrestler is thickened and keratinized. Osafune et al. reported that there is a measurable amount of bacterial flora in dohyo soil [26]. Thus, it is not uncommon for sumo wrestlers to suffer from cellulitis of the lower leg [17]. In addition, wrestlers are often overweight in order to increase their physical constitution, and they may suffer from diabetes at a young age [16, 17, 27]. Thus, it is necessary to consider the risk of infection during decision-making.

The three cases in the present study demonstrate the problem of treatment compliance; all three patients discontinued treatment of their own accord. Since they returned to competing in tournaments by their own judgment, complications such as nonunion, delayed union, and refractures occurred. Taking a rest may result in a lowered rank, rendering it difficult for the sumo wrestler to allow a sufficient duration of treatment. Therefore, the treatment of sumo wrestlers is controversial. In addition, even though a sumo wrestler may have some pain, they may not go to a hospital because they are not rested. Finally, sumo wrestlers potentially experience more trauma in the lower extremities, including Jones fractures, than in other body areas.

## 4. Conclusions

We experienced three cases of Jones fracture in sumo wrestlers. These cases demonstrate the difficulties of treating Jones fractures in sumo wrestlers. It is important to thoroughly inform sumo wrestlers of the treatment options and to decide the optimal treatment method for each patient.
